# *PICKLE RELATED 2* is a Neofunctionalized Gene Duplicate Under Positive Selection With Antagonistic Effects to the Ancestral *PICKLE* Gene on the Seed Transcriptome

**DOI:** 10.1093/gbe/evad191

**Published:** 2023-11-03

**Authors:** Gilles Dupouy, Ronan Cashell, Galina Brychkova, Reetu Tuteja, Peter C McKeown, Charles Spillane

**Affiliations:** Genetics and Biotechnology Lab, Agriculture & Bioeconomy Research Centre, Ryan Institute, University of Galway, Galway H91 REW4, Ireland; Genetics and Biotechnology Lab, Agriculture & Bioeconomy Research Centre, Ryan Institute, University of Galway, Galway H91 REW4, Ireland; Genetics and Biotechnology Lab, Agriculture & Bioeconomy Research Centre, Ryan Institute, University of Galway, Galway H91 REW4, Ireland; Genetics and Biotechnology Lab, Agriculture & Bioeconomy Research Centre, Ryan Institute, University of Galway, Galway H91 REW4, Ireland; Genetics and Biotechnology Lab, Agriculture & Bioeconomy Research Centre, Ryan Institute, University of Galway, Galway H91 REW4, Ireland; Genetics and Biotechnology Lab, Agriculture & Bioeconomy Research Centre, Ryan Institute, University of Galway, Galway H91 REW4, Ireland

**Keywords:** positive selection, reproduction, neofunctionalization, duplication, intrinsic disorder

## Abstract

The evolution and diversification of proteins capable of remodeling domains has been critical for transcriptional reprogramming during cell fate determination in multicellular eukaryotes. Chromatin remodeling proteins of the CHD3 family have been shown to have important and antagonistic impacts on seed development in the model plant, *Arabidopsis thaliana*, yet the basis of this functional divergence remains unknown. In this study, we demonstrate that genes encoding the CHD3 proteins PICKLE (PKL) and PICKLE-RELATED 2 (PKR2) originated from a duplication event during the diversification of crown Brassicaceae, and that these homologs have undergone distinct evolutionary trajectories since this duplication, with *PKR2* fast evolving under positive selection, while *PKL* is subject to purifying selection. We find that the rapid evolution of *PKR2* under positive selection reduces the encoded protein's intrinsic disorder, possibly suggesting a tertiary structure configuration which differs from that of PKL. Our whole genome transcriptome analysis in seeds of *pkr2* and *pkl* mutants reveals that they act antagonistically on the expression of specific sets of genes, providing a basis for their differing roles in seed development. Our results provide insights into how gene duplication and neofunctionalization can lead to differing and antagonistic selective pressures on transcriptomes during plant reproduction, as well as on the evolutionary diversification of the CHD3 family within seed plants.

**Significance**

PKL and PKR2 are two proteins from the same gene family participating in chromatin remodeling and gene expression control in plants, but which opposing effects during seed development on the establishment of final seed size. In this study, we investigate the evolution of PKL and PKR2 in land plants as well as their respective impact on whole genome expression. We show that PKR2 originates from a gene duplication of PKL early in the history of the Brassicaceae family, that its sequence changes faster than that of PKL and that it appears to confer an antagonistic effect on the expression of specific genes. Our results provide a basis for understanding the opposing effects of these genes during seed development in *Arabidopsis thaliana*.

## Introduction

Gene accessibility for transcription is linked to chromatin condensation and de-condensation which is regulated in a tissue-specific manner during the growth and development of multicellular eukaryotes, including during the establishment of their reproductive cell lineages (Batista and Köhler 2020). Two major protein complexes containing PolyComb (PcG) proteins and Trithorax (Trx)-like Chromodomain-helicase-DNA-binding (CHD) chromatin remodeler family proteins regulate many transcriptomic changes during development. In land plants (Embryophytes), the components of the PcG complexes have been well described, and form either type 1 or 2 PcG Repressive Complexes, PRC1 and PRC2, respectively (Grossniklaus et al. 1998; Kiyosue et al. 1999; Luo et al. 1999; Ohad et al. 1999; Köhler et al. 2003a; Köhler et al. 2003b). PRC1 and PRC2 can be recruited independently to gene targets to repress transcription, while also potentially establishing binding sites for each other to reinforce this repression (Mozgova and Hennig 2015). Trx-like CHD proteins, on the other hand, are ATP-dependent chromatin remodelers which function individually. They possess one or two chromodomains and can act synergistically or antagonistically with the PcG complexes depending of cellular context (Jacobs and Khorasanizadeh 2002; Nielsen et al. 2002; Murawska et al. 2008; Aichinger et al. 2011). For instance, the PcG genes *SWINGER* (*SWN*) and *EMBRYONIC FLOWER 2* (*EMF2*) are direct targets of the CHD3 protein PICKLE (PKL) in plant root tissues which acts upstream of the PRC complexes in which SWN and EMF2 participate (Aichinger et al. 2009).

The CHD family protein members possess a double chromodomain in their N-terminal regions, which specifically recognizes methylation marks on Histone H3 (Flanagan et al. 2005). Most members of this gene family also possess a pair of plant homeodomains (PHD) which preferentially recognize H3K4me3 (Wysocka et al. 2006). The CHD3 family protein (a class of chromatin remodeler members of the CDH subfamily II) comprises four members in *Arabidopsis thaliana*: PICKLE (PKL) and PICKLE-RELATED 1, 2, and 3 (PKR1, PKR2, and PRK3). Among these four, PKR2 appears to be most closely related to PKL, with PKR1 and PKR3 more distantly related (Ho et al. 2013). Importantly, PKR2 is not found among the CHD3 family proteins in animals, suggesting that the duplication of PKL and PKR2 may have occurred within land plants or the lineage leading to them. Despite their close relationship, PKL and PKR2 have been reported to play antagonistic roles in seed size determination in *A. thaliana*. On one hand, a knockout of *PKL* leads to a significant seed size increase associated with a decreased fertility, while on the other hand the knockout of *PKR2* can rescue *pkl* seed size and decreased fertility phenotypes, despite having no significant effect on seed size per se (Carter et al. 2016).

*PKR2* was shown to be under positive selection by Tuteja et al. (2019), but no focused molecular evolutionary analysis has been performed on *PKL* itself, nor on others members of the family. Gene duplication scenarios where one of the duplicates is fast evolving under positive selection (rather than evolving neutrally toward pseudogene outcomes), while the other one remains under purifying selection can be indicative of neofunctionalization of the fast-evolving duplicate (Gibson and Goldberg 2009; Sandve et al. 2018). In this study, we investigate the evolution of PKR2 and PKL within Embryophytes and the hypothesis of neofunctionalization of *PKR2* in comparison to *PKL* in Brassicaceae.

## Results

### PKR2 Originates From a Duplication of PKL During the Diversification of Brassicaceae

**Fig. 1.**
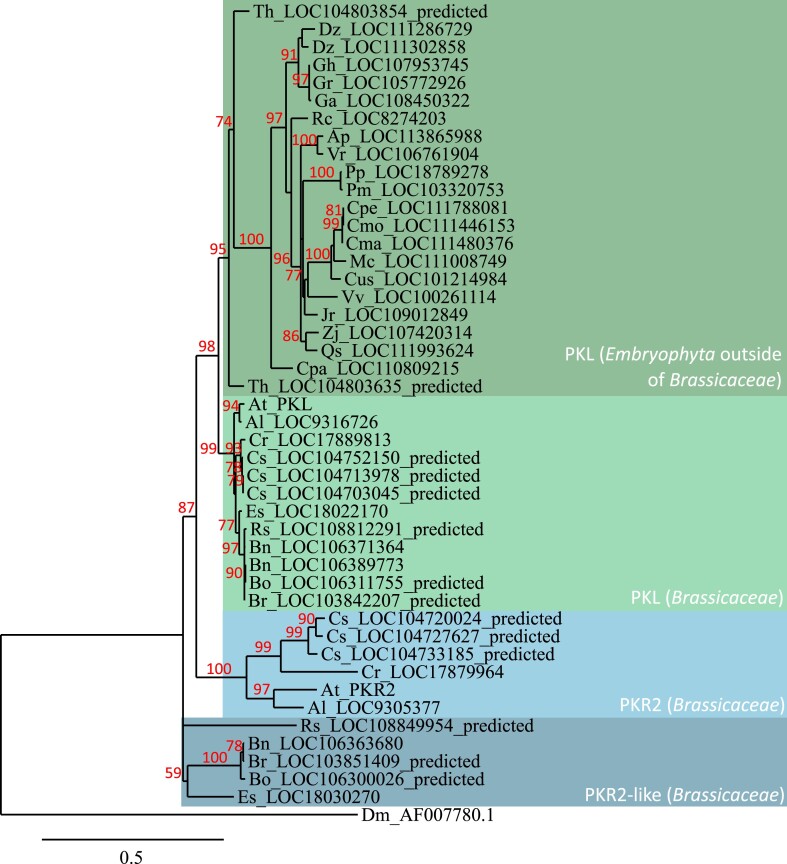
Evolution of PKL and PKR2 in high plants. Phylogenetic tree of PKL and PKR2 CHD3 chromatin remodelers in high plants rooted on *Drosophila melanogaster*. Bootstraps of high support branches are indicated as percentages. The tree was obtained using the SH-like maximum likelihood method. Each species is indicated thereafter with its initials (genome assembly versions used for this tree are displayed in parentheses). Ap: *Abrus precatorius* (v. 2018); At: *Arabidopsis thaliana* (TAIR10.1); Al: *Arabidopsis lyrata* (v2.1); Bn: *Brassica napus* (v2.0); Bo: *Brassica oleracea* (v1.0); *Br: Brassica rapa* (v1.3); Cma: *Cucurbita maxima* (v1.0); Cmo: *Cucurbita moschata* (v1.0); Cpa: *Carica papaya* (v1.0); Cpe: *Cucurbita pepo* (v2.0); Cus: *Cucumis sativus* (v3.0); Cs: *Camelina sativa* (v1.0); Cr: *Capsella rubella* (v1.1); Dm: *Drosophila melanogaster* (v1.0); Dz: *Durio zibethinus* (v1.0); Es: *Eutrema salsugineum* (v1.0); Ga: *Gossypium arboreum* (v1.0); Gh: *Gossypium hirsutum* (v1.1); Gr: *Gossypium raimondii* (v2.1); Jr: *Juglans regia* (v2.0); Mc: *Momordica charantia* (v2.0); Pm: *Prunus mume* (v1.0); Pp: *Prunus persica* (v2.1); Qs: *Quercus suber* (v1.0); Rc: *Ricinus communis* (v0.1); Rs: *Raphanus sativus* (v.1.0); Th: *Tarenaya hassleriana* (v1.0); Vr: *Vigna radiata* (v1.0); Vv: *Vitis vinifera* (v2.1); Zj: *Ziziphus jujuba* (v2.0).

To investigate the evolution of *PKL* and *PKR2* in land plants, high similarity protein sequences of PKL and PKR2 were identified using BLAST within Embryophyte genomes from the Phytozome and NCBI databases. Duplicates were removed and sequences aligned with PKL sequence from *Drosophila melanogaster* as an outgroup to generate a rooted phylogenetic tree (fig. 1); Sections of the sequence alignment are shown in supplementary figure S1, Supplementary Material online. Three main clades can be identified on the phylogenetic tree: PKL proteins within Brassicaceae, PKL proteins outside of Brassicaceae, and PKR2 proteins within Brassicaceae. This last clade is divided into two groups: PKR2 sequences as in *A. thaliana*, *A. lyrata*, *Camelina sativa* and *Capsella rubella*, and “PKR2-like” sequences as in *Brassica napus*, *Brassica oleracea*, *Brassica rapa*, *Eutrema salsugineum*, and *Raphanus sativus*. These results further indicate that PKR2 is specific to the Brassicaceae family and may have originated from a duplication of PKL prior to the diversification of crown Brassicaceae. The branch length indicates that PKR2 is evolving faster than PKL within the Brassicaceae. Protein sequence alignment shows that in comparison to PKL, PKR2 mostly lacks parts of the PHD zinc finger domain characteristic of the CHD3 family (supplementary figs. S1*a* and S2, Supplementary Material online) as well as a small portion of the SNF2-related N-terminal domain related to a loop structure of high disorder value (supplementary figs. S1*b* and S2, Supplementary Material online). Specific differences can be identified within these two locations between PKR2 and PKR2-like clades. While the PKR2 clade lacks portions of the PHD domain, the proteins from the “PKR2-like” clade lack this domain entirely. In contrast, the proteins from the “PKR2-like” clade lack a smaller portion of the SNF2-related N-terminal domain when compared to the proteins from the PKR2 clade. Protein disorder prediction with IUPRED3 suggests that this small portion of the SNF2 N-terminal domain corresponds to a peak region of high intrinsic disorder, as this peak is not present in PKR2 (supplementary fig. S2, Supplementary Material online).

### PKR2 is Under Positive Selection, While PKL is Under Purifying Selection

**Fig. 2.**
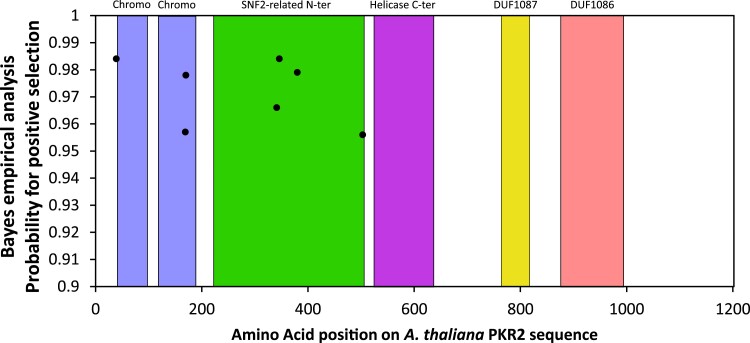
Sites under positive selection in PKR2. Amino acids sites of PKR2 under positive selection (*P* > 0.90). Each box represents the different domains of the protein as predicted by Pfam.

Our tests indicated positive selection within *PKR2* in *A. thaliana*, with significant χ^2^ tests comparing models with values of ω > 1, compared to those without. Bayes empirical Bayes posterior estimations of positive selection at specific sites in *PKR2* in *A. thaliana* identified 13 sites which had significant instances of base substitution (PAML output files available within Supplementary Material online). After sequence alignment verification (alignment file and resulting tree file available within Supplementary Material online) six of these amino acid positions were removed from further analysis as they preceded a deleted/poorly conserved region. The remaining seven amino acid positions were considered to have robust PAML evidence for being under positive selection (fig. 2). One of these amino acid sites is located on a loop at position 39 before the first chromo domain, just before the deleted PHD domain region, according to results obtained using Phyre2 and Alphafold (supplementary fig. S3*a*, Supplementary Material online) (Kelley et al. 2015; Jumper et al. 2021). Two amino acid sites are located within the second chromo domains at position 169 and 170, both forming a small predicted alpha helix linking two beta sheets (supplementary fig. S3*b*, Supplementary Material online). Finally, the remaining four amino acid sites are located within the SNF2-related N-terminal domain at positions 341 and 346 immediately after the deleted region from SNF2-related N-ter domain (supplementary fig. S3*c*, Supplementary Material online), and also at 380 (supplementary fig. S3*d*, Supplementary Material online) and 503 (supplementary fig. S3*e*, Supplementary Material online). The first two amino acid positions of these four are located within a small predicted alpha helix, the third in a predicted beta sheet, and the last in a small alpha helix.

### PKL and PKR2 Act Antagonistically on Seed Transcriptome

**Fig. 3.**
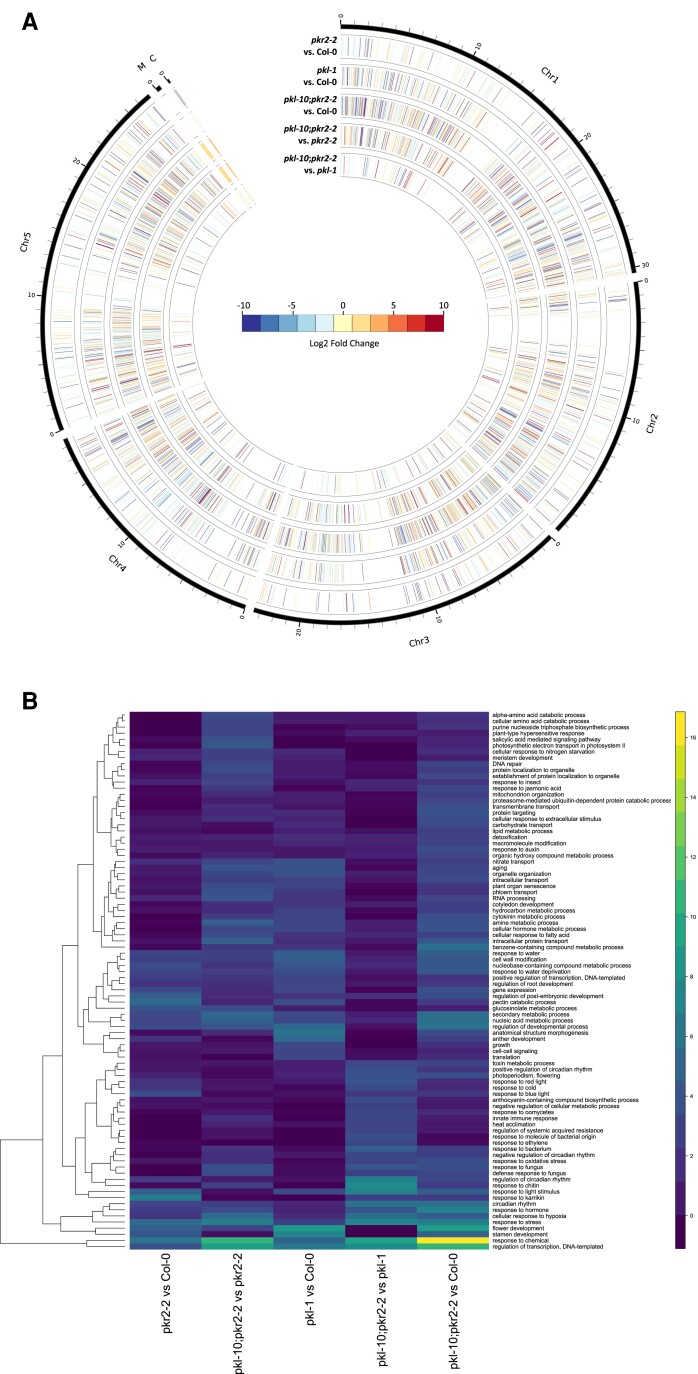
Differentially expressed genes (DEG). Genomic mapping of DEGs and GO enrichment in comparisons of four DAP seeds transcriptomes between *pkr2-2*, *pkl-1*, *pkr2-2;pkl-10* and WT Col-0. (*A*) Circular genome representation of DEGs observed between Col-0, *pkr2-2*, *pkl-1*, and *pkr2-2;pkl-10*. Each bar represents one gene at its position on its chromosome. Color scale represent the Log2 expression fold change for a DEG between samples. M, mitochondrion genome; C, chloroplast genome. (*B*) Heatmap dendrogram of the –Log10 of significant gene ontology terms *P*-value for DEGs sets between Col-0, *pkr2-2*, *pkl-1*, and *pkr2-2;pkl-10*.

To investigate possible transcriptome effects associated with the divergent functions of PKL and PKR2 on seed development, we used a suite of mutant lines (in Col-0 background) which have previously been shown to be null (knockout) mutants. These were *pkr2-2*, that is, SALK_115303 (Aichinger et al. 2009), *pkl-1* (Ogas et al. 1997) and *pkl-10,* that is GABI_273E06 (Zhang et al. 2012). We performed an RNA-seq on seeds from manually selfed *A. thaliana* plants of wild-type (WT) accession Col-0, compared to *pkr2-2* and *pkl-1* single mutants and a *pkr2-2;pkl-10* double mutant, in all cases sampling at the same seed development timepoint (four days after pollination). The RNA-seq analysis identified 391 differentially expressed genes (DEGs) between *pkr2-2* and Col-0, 1,017 DEGs between *pkl-1* and Col-0, 1,820 DEGs between *pkr2-2*;*pkl-10* and Col-0, 1,228 DEGs between *pkr2-2*;*pkl-10* and *pkr2-2*, and 282 DEGs between *pkr2-2*;*pkl-10* and *pkl-1* (fig. 3*a*).

To identify common and divergent transcriptome effects of *PKL* and *PKR2* on biological functions, we performed a gene ontology (GO) enrichment analysis. As expected, more GO terms were enriched in the DEGs between *pkl-1*and Col-0 than observed between *pkr2-2* and Col-0, corresponding to the higher number of DEGs in the latter. In both cases where the wild type was compared to each of the single mutants, DEGs were enriched in GO terms associated with responses to biotic and abiotic stresses, but some GO terms differed between the DEGs observed in the two mutants. While DEGs between *pkr2-2* and Col-0 were enriched in genes related to cell wall organization, development, metabolism, and transcription, those between *pkl-1* and Col-0 were enriched in genes related to cell–cell signaling, organellar organization, and translation (fig. 3*b*). DEGs identified in the comparison between the double mutant *pkr2-2*;*pkl-10* and Col-0 showed similar enrichments to those found between the single mutants and Col-0. However, some GO terms such as circadian rhythm, photosynthesis, ageing, and response to hormones were specifically found in the double mutant compared to Col-0.

To test the transcriptome effects on gene function between single and double mutants of *PKL* and *PKR2*, we also compared the GO terms in the DEGs identified in the double mutants against each of the single mutants. The DEGs dysregulated between *pkr2-2*;*pkl-10*; and *pkr2-2* displayed similar GO term enrichment to those observed between *pkl-1* and Col-0 (i.e., DEGs specific to *PKL*), while DEGs dysregulated between *pkr2-2*;*pkl-10* and *pkl-1* displayed similar GO term enrichment to those between *pkr2-2* and Col-0 (i.e., DEGs specific to *PKR2)*. However, GO terms for circadian rhythm, photosynthesis and response to hormone were also enriched in the DEG sets overlapping between *pkr2-2*;*pkl-10* and *pkr2-2* and between *pkr2-2*;*pkl-10* and *pkl-1*. Overall, our RNA-seq analysis indicates that the *pkl* and *pkr2* mutants tested have some seed transcriptome DEGs which are independent of each mutant, while also having an overlapping set of DEGs.

For each DEG set, we also investigated which biological pathways were dysregulated (supplementary fig. S4, Supplementary Material online). Cell wall plasticity was found to be enriched between *pkr2-2* and Col-0, with an upregulation of the pectin lyase-encoding genes AT1G67750 and AT3G07010, possibly indicating cell wall loosening during seed expansion. The same pathway was enriched between *pkl-1* and Col-0 and dysregulated in the same direction (upwards). In addition to the two genes mentioned above, the pectin lyase-encoding gene AT3G01270 was found to be upregulated in in *pkl-1* compared to Col-0, while AT2G45220 [encoding a pectin methylesterase (PME) inhibitor] was downregulated. Cell wall plasticity genes were also dysregulated in *pkr2-2;pkl-10* in comparison to Col-0 (upregulation of AT3G07010 and AT1G02810, the latter encoding a PME inhibitor), while a dysregulation of secondary metabolite pathways was also found with upregulation of lysine-ketoglutarate reductase-encoding *LKR* and downregulation of CTP synthase CTPS5-encoding *AT2G34890*. The comparison between *pkr2-2*;*pkl-10* and *pkr2-2* also showed an enrichment in regulation of cell wall plasticity pathway (as also observed for *pkl-1* and Col-0), with the downregulation of *AT2G45220* and *AT3G09340* (pectin lyase) and the upregulation of *AT3G10720* (PME inhibitor). None of the pathways described above were enriched between *pkr2-2;pkl-10* and *pkl-1.* However, in comparison between these two mutant lines, the fatty acid biosynthesis pathway was dysregulated with downregulation of the gene AT4G17470 encoding an alpha/beta hydrolase.

**Fig. 4.**
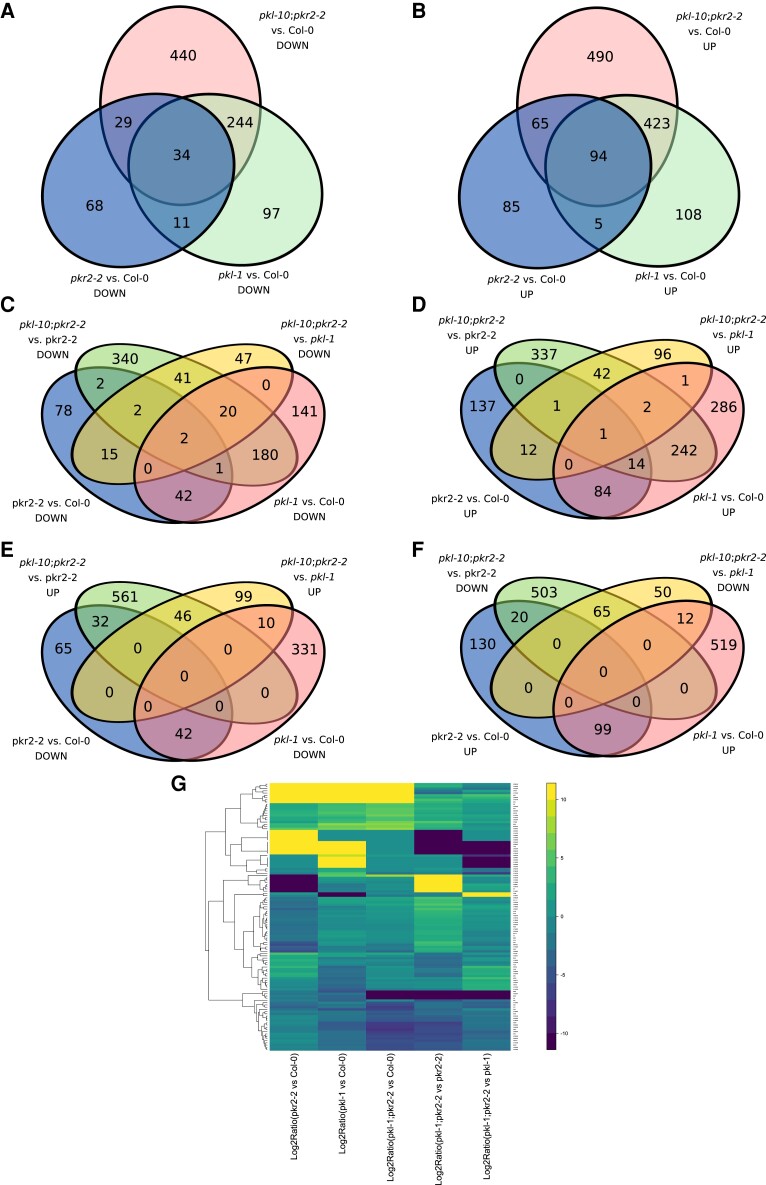
Overlaps of differentially expressed genes (DEG). Overlaps and clusters of DEGs between the different subsets calculated between four DAP seed transcriptomes of *pkr2-2*, *pkl-1*, *pkr2-2;pkl-10*, and WT Col-0 4 DAP seeds. (*A*–*F*) Venn diagram of downregulated (*A* and *C*) and upregulated (*B* and *D*) DEGs between *pkr2-2*, *pkl-1*, and *pkr2-2;pkl-10* in comparison to WT Col-0 (*A* and *B*) and between *pkr2-2* and *pkl-1* compared to Col-0 and *pkr2-2;pkl-10* compared to *pkr2-2* and *pkl-1* (*C* and *D*). (*E* and *F*) Venn diagram of oppositely dysregulated genes between *pkr2-2* and *pkl-1* compared to Col-0 and *pkr2-2;pkl-10* compared to *pkr2-2* and *pkl-1.* (*G*) Heatmap dendrogram of overlapping DEGs between *pkr2-2*, *pkl-1* and *pkr2-2;pkl-10* and WT Col-0.

The overlap between the different DEG comparison sets was also analyzed (fig. 4). Significant overlap of DEGs was found between all three mutants (i.e., *pkl-1, pkl-10, pkr2-2*) in comparison to Col-0, whether upregulated or downregulated (fig. 4*a* and *b*). In this regard, 34 downregulated and 94 upregulated DEGs were found to be common to all three mutants (when compared to Col-0) and were enriched for GO terms such as development, response to abiotic stress and cell wall organization (with common upregulation of four pectate lyase genes). In addition, 203 down- and 259 upregulated DEGs were common between the *pkr2-2;pkl-10* versus *pkr2-2* and *pkl-1* versus Col-0 sets, and 19 down- and 14 upregulated DEGs wee common the between *pkr2-2;pkl-10* versus *pkl-1* and *pkr2-2* versus Col-0 sets. Some overlap was also found between the *pkr2-2;pkl-10* versus *pkr2-2* and *pkr2-2* versus Col-0 (7 down- and 16 upregulated DEGs) sets, and between *pkr2-2;pkl-10* versus *pkl-1* and *pkl-1* versus Col-0 (22 down- and 4 upregulated genes). These last two overlaps of DEGs include transcription factors, cell wall remodelers, circadian rhythm, and photosynthesis-associated genes. Finally, we found an overlap of oppositely dysregulated DEGs (i.e., upregulated in one set, while downregulated in the other set) between *pkr2-2;pkl-10* versus *pkl-1* and *pkl-1* versus Col-0 on the one hand (10 downregulated and 12 upregulated DEGs in *pkl-1* vs. Col-0), and the same genes oppositely dysregulated DEGs in the other set). Similarly, the comparisons of *pkr2-2;pkl-10* versus *pkr2-2* with *pkr2-2* versus Col-0 identified a set of oppositely dysregulated DEGs (32 downregulated and 20 upregulated DEGs in pkr2-2 vs. Col-0 and oppositely dysregulated in the other set) (fig. 4*e* and *f*). The genes oppositely dysregulated in *pkr2-2;pkl-10* versus *pkr2-2* and *pkr2-2* versus Col-0 sets were enriched for genes involved in ubiquitination and cell wall components. Genes oppositely dysregulated in *pkr2-2;pkl-10* versus *pkl-1* and *pkl-1* versus Col-0 were enriched for genes involved in abiotic stress response.

The heatmap dendrogram (fig. 4*g*) demonstrates that genes commonly dysregulated between *pkr2-2;pkl-10* versus *pkr2-2* and *pkr2-2* versus Col-0 tend to also be dysregulated in the same direction (i.e., either UP or DOWN) in *pkr2-2;pkl-10* versus *pkl-1* and *pkl-1* versus Col-0. We also observed that the oppositely dysregulated genes between either *pkr2-2;pkl-10* versus *pkr2-2* and *pkr2-2* versus Col-0 sets or *pkr2-2;pkl-10* versus *pkl-1* and *pkl-1* versus Col-0 sets are not significantly dysregulated in the *pk2-2; pkl-10* versus Col-0 set, possibly suggesting that these are dysregulated by each single *pkr2-2* or *pkl-1* mutant allele, but return to WT levels when both mutations are combined. This demonstrates that the antagonistic opposite effects at the functional genetic level fo*r pkl* and *pkr2*, are recapitulated at the transcriptome level where antagonistic effects are observed on a subset of the seed transcriptome DEGs identified in this study.

## Discussion

### *PKR2* Has Diverged From *PKL* Within Brassicaceae and Undergone Reduction in Protein Disorder Under Positive Selection

The evolution of PKR2 is unique within the CHD3 chromatin remodeler family. The three clades represented by *PKL*, *PKR1*, and *PKR3* are conserved between animals and plants, whereas *PKR2* is the only plant-specific gene of the family and has been until now classified within the PKL clade for its close resemblance with PKL despite the lack of a PHD domain (Ho et al. 2013). We demonstrate here that *PKR2* originated from a duplication of *PKL* during the early divergence of Brassicaceae, which is in agreement with Trujillo et al. (2022), and that *PKR2* diverged from *PKL*. Our finding that *PKR2* is under positive selection (while *PKL* is not) and that *PKL* seems to be relatively well-conserved across Embryophytes supports the possibility of a neofunctionalization of *PKR2*. Gene neofunctionalization events are often associated with a gene duplication followed by one of the duplicates fast diverging under selection, while the other remains under purifying selection (Gibson and Goldberg 2009; Sandve et al. 2018). Indeed, PKL function is essential in plants for normal cell fate determination through the maintenance of H3K27me3 homeostasis (Ogas et al. 1997, 1999; Carter et al. 2016; Carter et al. 2018), which is consistent with the purifying selection which acts on the *PKL* coding sequence.

The fact that most amino acid positions under positive selection in the PKR2 sequence are located in either the Chromo or SNF2-related N-terminal domains could signal their importance for a newly acquired function of PKR2, which would make sense given their roles in H3 tail recognition. Positive selection in the ATPase SNF2-related N-terminal domain is more surprising due to the highly conserved nature of ATPases (Linder et al. 2004; Ho et al. 2013), although rapid evolution here could be related to the loss of the highly disordered loop structure in the middle of the SNF2-related N-terminal domain. Indeed, 3D structure prediction of PKL and PKR2 suggests that the loss of this loop causes an important change to protein folding, especially in the C-terminal regions (supplementary fig. S2*c* and *d*, Supplementary Material online). In PKL, the loop structure is closely adjacent to the C-terminus. In contrast, the loss of this loop in PKR2 seems to simplify the structure which is no longer in close proximity with the C-terminus. This decreases disorder at that location, leading to the observed suppression of the associated disorder peak in the IUPRED prediction (supplementary fig. S2*b*, Supplementary Material online). Furthermore, the apparent release of the C-terminal end of the protein in PKR2 is associated with a smoothing of the predicted disorder signal. The modifications of the PKR2 protein likely result in an overall reduction in the level disorder within the protein structure (in comparison with PKL).

Apart from N39 and P503, all the amino acid sites under positive selection are predicted to stabilize the 3D structure of the protein through hydrogen bonding, again suggesting that *PKR2* may be evolving to reduce disorder within the protein. This would agree with hypotheses that disorder-to-order transitions are features of both subfunctionalization and neofunctionalization after gene duplication events (Ahrens et al. 2016; Ahrens et al. 2017). According to this mechanism, some conformations newly obtained by mutations can be stabilized by novel protein interactions and thus be selected through the course of evolution, followed by a loss of disorder to stabilize the new conformation (Schlessinger et al. 2011; Mosca et al. 2012; Pechmann and Frydman 2014). The reduction of disorder in PKR2 could thus suggest that it is adjusting to a new conformation that differs from that of the ancestral PKL. As intrinsically disordered regions can affect protein–protein interactions in intra-nuclear compartments (Meng et al. 2015), the lack of these regions in PKR2 could be associated with a different subnuclear localization of PKR2 in comparison to PKL.

### Divergent and Antagonistic Roles of *PKL* and *PKR2* in Transcriptome Regulation

In Arabidopsis, *PKL* and its diverged homolog *PKR2* have different roles in control of seed development, which are consistent with models of neofunctionalization. However, their respective roles in transcriptome regulation have not been investigated. Here, we perform RNA-seq and reveal that a knockout mutant allele of *PKR2* (i.e., *pkr2-2*) causes less dysregulation of the transcriptome than *PKL* loss of function alleles (*pkl-1* and *pkl-10*). This observation at the seed transcriptome level is consistent with functional genetics of these mutants, as only *pkl* mutants display visible changes to seed development (Carter et al. 2016). Surprisingly, although the *pkr2-2;pkl-10* double mutant rescue the seed phenotype of a *pkl-10* mutants, we found more DEGs in the double mutant than in the two single mutants combined (i.e., DEGs of both *pkr2-2* and *pkl-10*). This suggests that PKL and PKR2 do not directly counteract each other's effects but could act antagonistically on the seed size phenotype via different genes and pathways. On the other hand, we identify the DEGs which are specific to either *pkl-1* or *pkr2-2*, and whose expression reverted to WT levels in the double mutant. These DEGs were enriched in for genes encoding ubiquitin-protein transferases or cell wall components, indicating that some directly antagonistic effects between *PKL* and *PKR2* could be observed in protein degradation pathways and cell wall organization.

Our analysis of pathway dysregulation further supported this, identifying four pectin lyase genes and three PME inhibitor genes within the DEGs. These are known to promote or restrict cell wall loosening during plant tissue growth (Marín-Rodríguez et al. 2002; Levesque-Tremblay et al. 2015). Four of the five lyases were upregulated in the mutants, while one PME inhibitor was downregulated in *pkl-1* and *pkr2-2;pkl-10* and the others were upregulated in *pkr2-2*, *pkl-1*, and *pkr2-2;pkl-10*. Hence, the increase in RNA expression levels of pectin lyases could help explain the increased seed size in *pkl-1*, with a compensatory increase of PME inhibitor activity counterbalancing this following mutation of *pkr2*. The molecular mechanisms by which PKL and PKR2 might control these cell wall genes, whether directly or indirectly, remain to be determined. Investigation of protein–protein and protein–DNA interactions for PKL and PKR2 could indicate whether these are due to direct or indirect effects of PKL versus PKR2 binding to chromatin. Further investigations of the role of PKL in transcriptome regulation in flowering plants outside of the Brassicaceae could identify the basal role in the absence of presence of PKR2.

## Conclusions

In this study, we demonstrate that *PKR2* originated from a duplication of *PKL* in the Brassicaceae linage and has subsequently evolved under positive selection with likely neofunctionalization. The PKR2 amino acids under positive selection are located within chromatin- and ATP-interacting domains and change PKR2 protein structure toward a reduction of its intrinsic disorder. The functional effects of these amino acids on the structure of PKR2 compared to PKL remain unexplored, especially in terms of interaction with histones. We conclude that the *PKL*/*PRK2* system provides a valuable model understanding the evolution of gene duplicates (including neofunctionalization), antagonistic molecular networks, and their ultimate functional effects on seed development.

## Materials and Methods

### Plant Material and Growth Conditions

*Arabidopsis thaliana* seeds were surface sterilized with Chlorine gas (3:1 bleach:hydrochloric acid in a bell jar for 1 h). Seeds were germinated on 0.5 × Murashige and Skoog (MS) medium (Murashige and Skoog, 1962) containing 1% w/v sucrose and 0.8% w/v agar, and grown in a percival tissue culture cabinet under a 16:8 h light:dark (21 °C/18 °C) regime (Boyes et al. 2001) until they were transferred to soil [five parts Westland compost (Dungannon, N. Ireland): 1 part perlite: 1 part vermiculite]. Plants were grown in chambers under fluorescent lamps at 200 μmol m^−2^ s^−1^ with the same photoperiod.

All knockout mutants were in Col-0 background: *pkr2-2*, that is SALK_115303 (Aichinger et al. 2009), *pkl-1* (Ogas et al. 1997) and *pkl-10,* that is GABI_273E0681 (Zhang et al. 2012). Primers used to genotype *pkr2-2* are 5′-GGGGAGGAGTATCTGGTGAAG-3′ (forward), 5′-ATTTTGCCGATTTCGGAAC-3′ (reverse wild type) with the SALK specific reverse primer 5′-ATTTTGCCGATTTCGGAAC-3′. The *pkl-1* mutant was genotyped as published by Ogas et al. (1997) using 5′-AGCATTCTGTGCCCAACTGA-3 (forward), 5′-CAATTCGCTCGTACTGCCATTAC-3 (reverse wild type) and 5′-CAATTCGCTCGTACTGCCATTAT-3′ (reverse mutant). The *pkl-10* mutant was genotyped using 5′-TGCGGTTTGTTTCTCCTCTCG-3′ (forward), 5′-TGTTTCAGAGACCCAAAATCGTG-3′ (reverse wild type) and the GABI specific reverse primer 5′-ATATTGACCATCATACTCATTGC-3′ (supplementary table S1, Supplementary Material online).

### Plant DNA Extraction

Plant genomic DNA was extracted using 20 mg of rosette leaf which was grinded with glass beads and incubated in DNA extraction buffer (200 mM of Tris–HCl pH 7.5, 250 mM of NaCl, 25 mM of EDTA, 0.5% of SDS) for 10 min at 60 °C. Samples were next mixed with equal volumes of ice-cold isopropanol (1:1) and DNA was precipitated at −20 °C for 10 min followed by a centrifugation. Precipitated DNA was washed once with 70% v/v ethanol, left over to dry, re-suspended in water, and incubated at 60 °C for 10 min.

### RNA Extraction and Sequencing

RNA was extracted from snap frozen seeds from at least 30 siliques per replicate collected at 3 DAP according to the protocol described by Allorent et al. (2010), with some modifications. In particular, seeds were homogenized in 2 ml Eppendorf tubes using a plastic reusable pestle and mixed with 1 ml RNA extraction buffer as described by Oñate-Sánchez and Vicente-Carbajosa (2008). The protocol continued as described (Allorent et al. 2010). DNase treatment was performed on 1 μg of crude RNA using the DNase I amplification grade kit (Invitrogen, UK), followed by clean-up and concentration for RNA-seq using RNA clean and concentrator kit from Zymo Research (Cambridge Bioscience, UK). RNA integrity was assessed by agarose gel electrophoresis, and at least 400 ng of RNA per sample were used for mRNA sequencing (Novogene, Cambridge, UK).

### Identification of PKR2 and PKL Homolog Sequences in Embryophytes

For each gene, an initial search was performed by using the *A. thaliana* sequences as query for a BLAST search (Mount 2007) within Embryophyte genomes from the Phytozome (version 13) and NCBI Refseq databases. Resulting sequences were compiled and aligned using MUSCLE [version 3.5, Edgar (2004)] on the phylogeny platform using default parameters (16 iterations maximum). Phylogeny was established using PhyML [version 3.0, Guindon et al. (2010)] with an SH-like approximate likelihood test, and the tree was build using TreeDyn [version 196, Chevenet et al. (2006)] to root sequences on the PKL homolog sequence from *D. melanogaster*. Disorder prediction of PKR2 and PKL in *A. thaliana* was performed using Iupred3 (Erdős et al. 2021).

### Testing for Positive Selection

Tests for positive selection were performed on a subset of 22 PKL and PKR2-like sequences, and PKR2 homologs to identify instances of positive selection within *A. thaliana*. The retrieved protein and cDNA sequences were aligned using Clustal Omega, followed by PAL2NAL to convert the alignment. RAxML was used to fit a maximum likelihood tree using this subset of sequences to determine not only topology but also branch lengths.

Positive selection tests were performed using the CodeML program within the PAML program. The analyses were performed using the branch-site selection model 2a, such that assessment of the model is carried out against a null model wherein ω (the potential rates of dn/ds) are fixed at 1 in comparison to a model without such fixation, and assessment of the alternate model's significance is carried out using a χ^2^ test. Multiple alternate models with different starting values for ω were used to address potential bias. Individual codons were considered as under positive selection subject to: 1) a significant difference in the null and alternate models, 2) a significant estimation of posterior probability using Bayes empirical Bayes, and 3) confirmation that the significant codon is in a conserved region of the PKL/PKR2-like proteins.

### RNA Sequencing and Bioinformatics Analysis

RNA-seq was performed on two biological replicates per sample, 20 million reads depth and pair end read. Bioinformatic analysis was performed sing the Galaxy servers (Afgan et al. 2018). Low-quality sequences with a PHRED Quality < 30 were removed using Fastp [Ver. 0.20.1, Chen et al. (2018)]. Resulting high-quality RNA sequences were mapped to reference genome TAIR10.1 using TopHat [Ver. 2.1.1, Kim et al. (2013)]. Cufflinks [Ver. 2.2.1.3., Trapnell et al. (2010)] was used to calculate gene relative expression using the fragment per kilobase per million (FPKM) method and DEGs were determined using geometrical method and negative binomial method for dispersion estimation (Poisson law). DEGs were considered as truly differentially expressed with a *q*-value < 0.05 and a Log2 fold change >2. Venn diagrams and heatmaps were generated using R-studio (Ver. 3.5.1) using the VennDiagram and ViridisLite packages. Gene ontology enrichment was performed using the Panther Algorithm (Mi et al. 2013) and dysregulated pathway enrichment with ShinyGAM [version 0.99.5-8-gec900f3, Sergushichev et al. (2016)].

Validation of RNA-seq results were done by RT-qPCR. Generation of cDNAs was done on previously treated crude RNA using the Superscript III First Strand Synthesis kit (Invitrogen, UK), according to manufacturer instructions. The RT-qPCR was performed using PowerUp SYBR green Master Mix (Applied BioSystem, UK) and corresponding primers (supplementary table S2, Supplementary Material online). Expression of target genes was normalized to *EF-1*α housekeeping gene (Wang et al. 2014) and relative expression was calculated in reference to a control sample using the Livak method. Primers are summarized in supplementary table S2, Supplementary Material online.

## Supplementary Material

Supplementary data are available at *Genome Biology and Evolution* online (http://www.gbe.oxfordjournals.org/).

## Supplementary Material

evad191_Supplementary_Data

## Data Availability

The data underlying this article are available in the article and in its Supplementary Material online. The Raw files and DEGs lists for the RNA-seq experiments are available publicly on the NCBI Gene Expression Omnibus under the identifier GSE236025.
