# Supplementary material for: PICKLE RELATED 2 is a Neofunctionalized Gene Duplicate Under Positive Selection With Antagonistic Effects to the Ancestral PICKLE Gene on the Seed Transcriptome
Source: Genome Biol Evol. 2023 Nov 3;15(11):evad191. doi: 10.1093/gbe/evad191 (PMC10630071; doi:10.1093/gbe/evad191)
Supplement: evad191_Supplementary_Data [file evad191_supplementary_data.zip › Dupouy_et_al_Supplementary_Materials_120923_Final.docx]

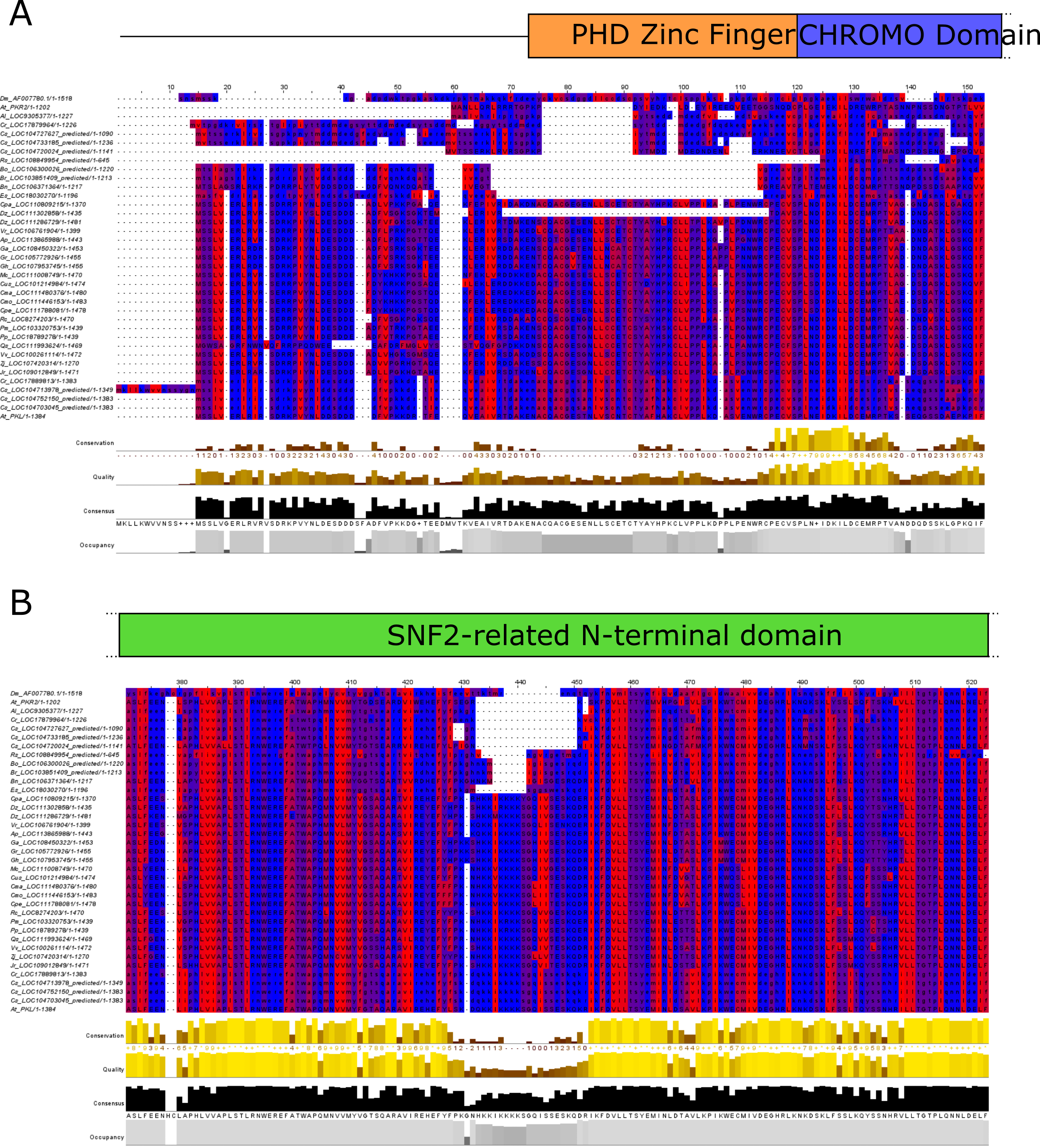


## Supplementary Figure S1: Alignment of PKL and PKR2 sequences from Embryophytes.

**A**: PhD zinc finger from PKL is absent from PKR2 sequence. **B**: A portion of the DEAD-like helicase superfamily domain is missing from PKR2. Amino acids are coloured in accordance with their hydrophobicity. Blue: Hydrophilic amino acid; Red: Hydrophobic amino acid.


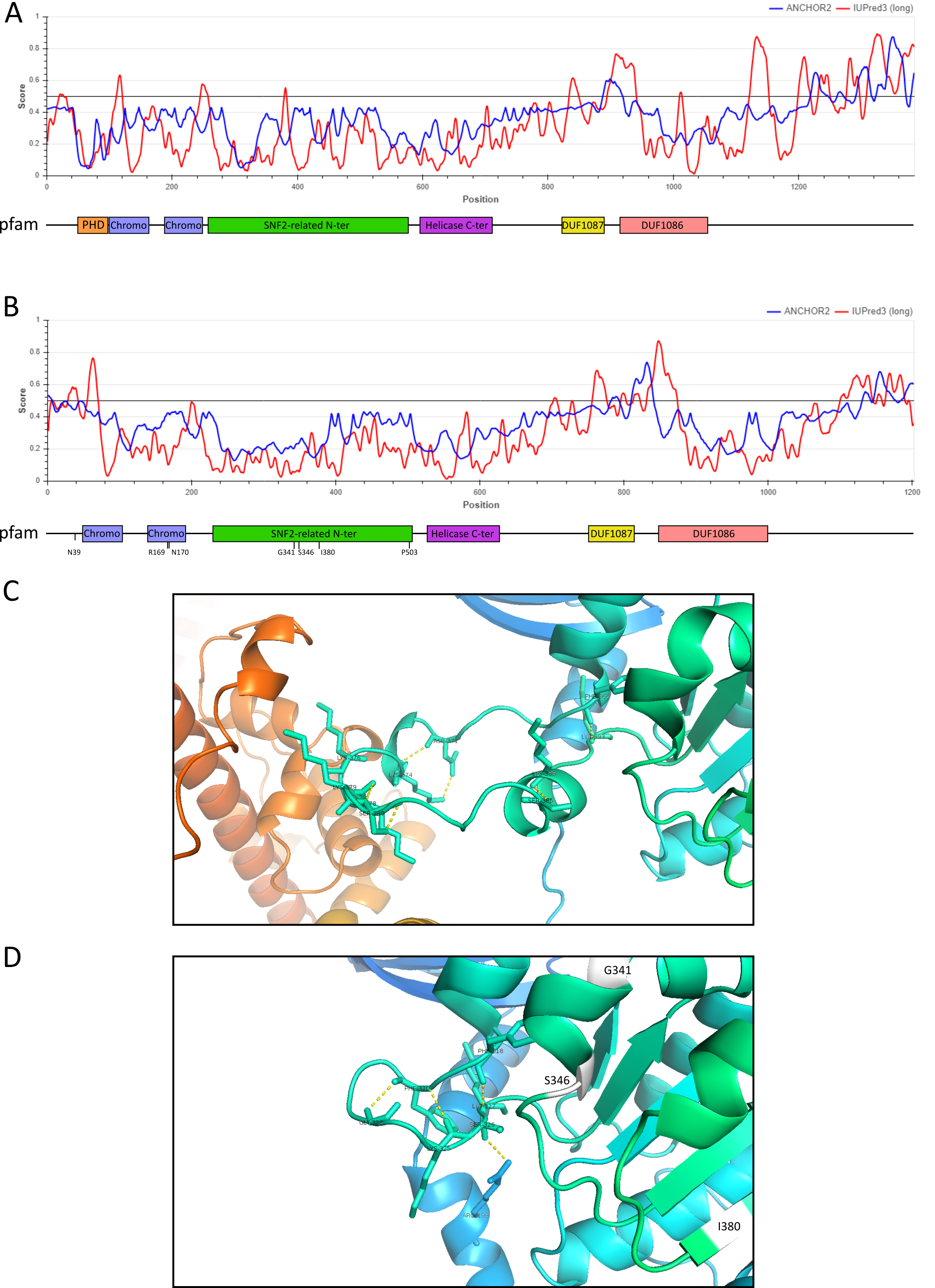


## Supplementary Figure S2: PKL and PKR2 intrinsic disorder prediction.

IUPRED3 and ANCHOR2 disorder prediction in PKL (**A**) and PKR2 (**B**) proteins. Loss of the PHD domain in PKR2 corresponds to the loss of a low disorder section, while the loss of a small loop section from the SNF2-related domain corresponds to the loss of a high disorder peak. The release of the C-terminal tail in PKR2, previously interacting with the SNF2-related domain small loop section in PKL seems to result in a smoothening of the disorder signal. Residues under positive selection are annotated under the Pfam obtained map of protein domains for PKR2 (B). **C and D**: Alphafold prediction of PKL (**C**) and PKR2 (**D**) NSF2-related N-terminal domain loop structure. Residues under positive selection in *PKR2* sequence are annotated and highlighted in grey (D).


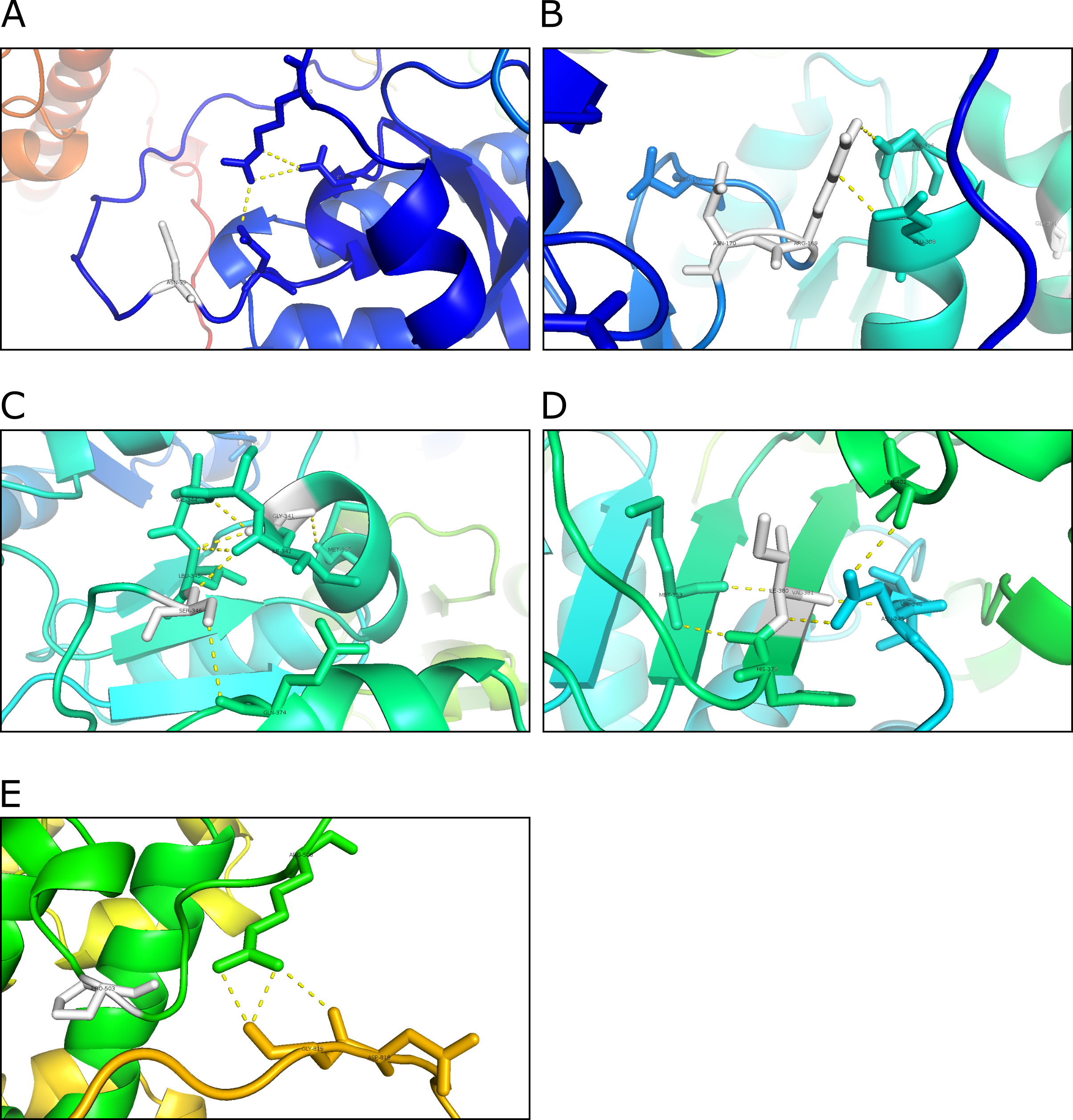


## Supplementary Figure S3: Prediction of intra-protein interaction of amino acid residues under positive selection in PKR2

Alphafold 3D prediction in PKR2 of intra-protein interactions of sites under positive selection (indicated in white). **A**: N 39 is not contributing to any higher order 3D structure, but is located near an inter-loop hydrogen bond. **B**: R 169 forms a double hydrogen bond with D 306 and E 308 from nearby alpha helix. N 170 is not predicted to form any non-covalent bond. **C**: G 341 forms hydrogen bonds with M 337, V 344 and L 345, which contribute to an alpha helix structure. S 346 is at the start of the alpha helix and forms hydrogen bonds with I 342. S 346 also forms a hydrogen bond with Q 314, maintaining cohesion between the two neighbouring alpha helices. **D**: I 380 forms a double hydrogen bond with N 245 and V 246 and participates in the cohesion of four neighbouring beta sheets. **E**: P 503 is not contributing to any higher order 3D structure, but is located near an inter-loop hydrogen triple hydrogen bond between R 506 and D 818 and G819.

## Supplementary Figure S4: Major dysregulated pathways in DEG subsets of 4 DAP seed transcriptome differences between Col-0, *pkr2-2*, *pkl-1* and *pkr2-2;pkl-10*.


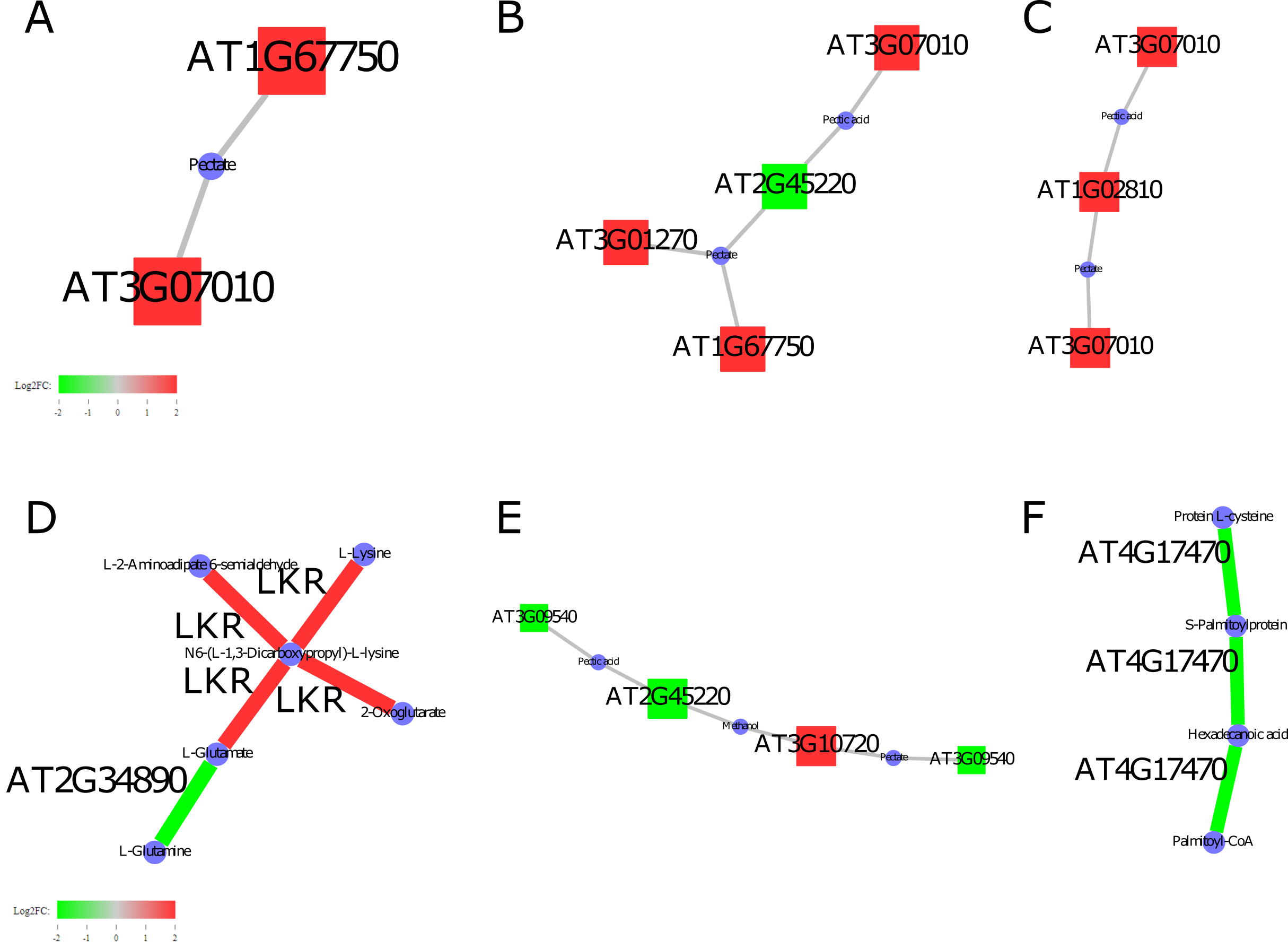


Significantly dysregulated pathways between Col-0, *pkr2-2*, *pkl-1* and *pkr2-2;pkl-10*. **A**: Dysegulation of genes encoding proteins associated with increase of cell wall loosening activity in *pkr2-2*, in comparison to Col-0. **B**: Dysegulation of genes encoding proteins associated with increase of cell wall loosening activity in *pkl-1*, in comparison to Col-0. **C**: Up-regulation of mRNA of genes encoding pectin lyases and pectin methylesterase inhibitors in *pkr2-2;pkl-10,* in comparison to Col-0. **D**: Decrease in mRNA levels of genes encoding CTP synthase and increase mRNA expression levels of genes encoding lysine-ketoglutarate reductase in *pkr2-2;pkl-10*, in comparison to Col-0. **E**: Dysegulation of genes encoding proteins associated with decrease of cell wall loosening activity in *pkr2-2;pkl-10,* in comparison to *pkr2-2*. **F**: mRNA expression levels of genes encoding thioesterases in *pkr2-2;pkl-10*, in comparison to *pkl-1*.


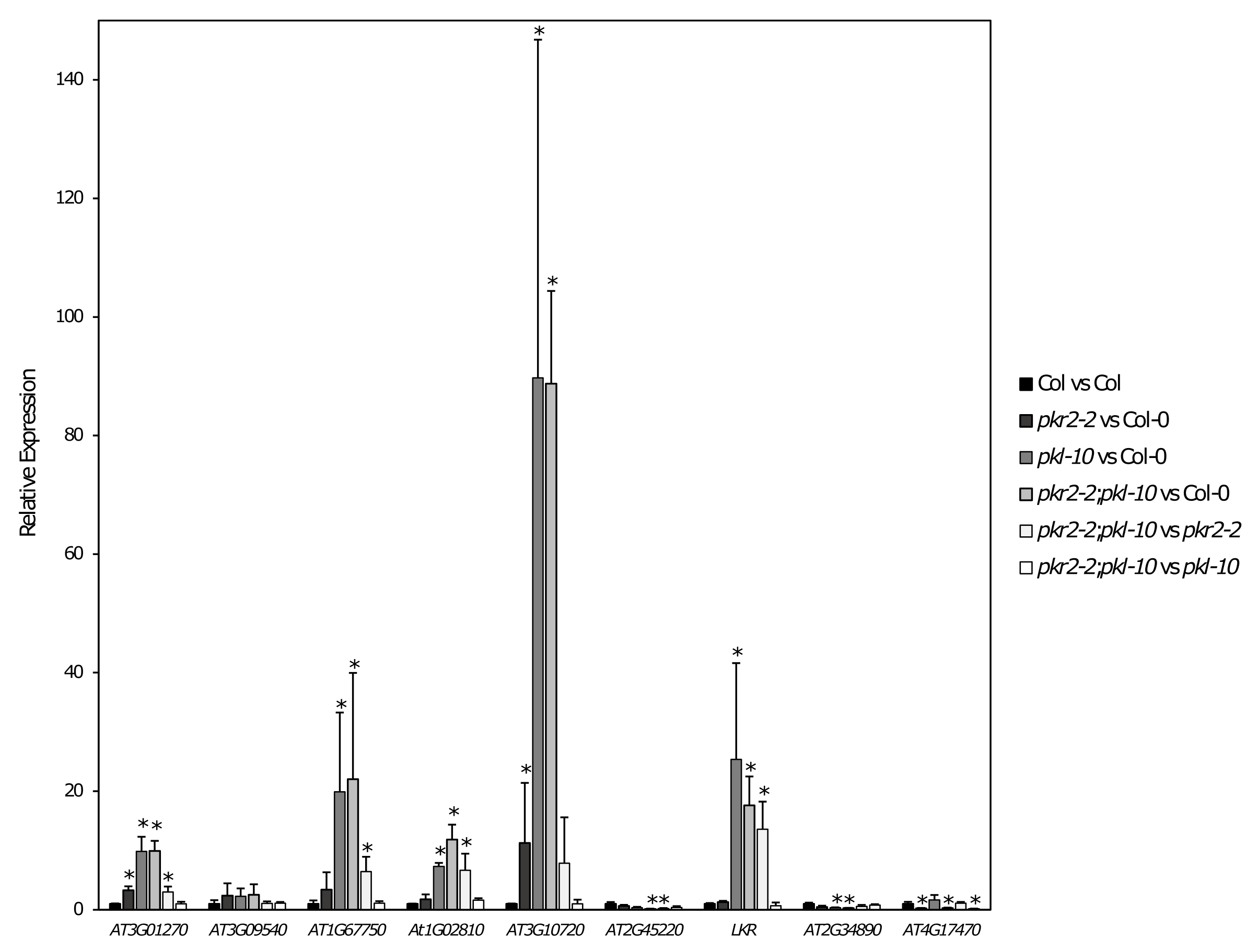


## Supplementary Figure S5: RT-qPCR verifications of target gene of interest identified from RNAseq.

Relative expression of gene targets. Relative gene expression of each gene is normalized with housekeeping gene *EF1α* expression and compared to a control sample using the Livak method. Stars indicate gene expression fold changes that are statistically significant according to Student test (p-value < 0.05).

| **Line** | **Primer** | **Sequence** |
| --- | --- | --- |
| ***pkr2-2*** | Forward | 5'-TTCTGATTTTTCAACCGATGG-3' |
|  | Reverse (WT) | 5'-GGGGAGGAGTATCTGGTGAAG-3' |
|  | Reverse (mutant) | 5'-ATTTTGCCGATTTCGGAAC-3' |
| ***pkl-1*** | Forward | 5'-AGCATTCTGTGCCCAACTGA-3' |
|  | Reverse (WT) | 5'-CAATTCGCTCGTACTGCCATTAC-3' |
|  | Reverse (mutant) | 5'-CAATTCGCTCGTACTGCCATTAT-3' |
| ***pkl-10*** | Forward | 5'-TGCGGTTTGTTTCTCCTCTCG-3' |
|  | Reverse (WT) | 5'-TGTTTCAGAGACCCAAAATCGTG-3' |
|  | Reverse (mutant) | 5'-ATATTGACCATCATACTCATTGC-3' |

## **Supplementary Table S1:** Genotyping primers for *pkr2* and *pkl* mutants.

| **Gene** | **Sense** | **Sequence** |
| --- | --- | --- |
| ***At3g01270*** | Forward | 5’-CACGAGCGTCATTACGACAAC-3’ |
|  | reverse | 5’-CTTTTCCTTTTCCCTTGCCCC-3’ |
| ***At3g09540*** | Forward | 5’-GCTGGTGTCGAATCTCAGATACAT-3’ |
|  | Reverse | 5’-CTAAGACACGATTTGGCTCCG-3’ |
| ***At1g02810*** | Forward | 5’-ACGGTGGTCACCGGAAATAG-3’ |
|  | Reverse | 5’-AGAAGATTGAGAAATCTGCGCC-3’ |
| ***At3g10720*** | Forward | 5’-ACGCACTTAACCGTAACCTGA-3’ |
|  | Reverse | 5’-CCCCTAAGTTCCGGCTAGTTTT-3’ |
| ***LKR*** | Forward | 5’-GGCAGAGCTGGTCTTGTTGA-3’ |
|  | Reverse | 5’-TAATGGCAGTCCCTGGCTTG-3’ |
| ***At2g34890*** | Forward | 5’-TCGTGTGATCTCGAAAAGACCA-3’ |
|  | Reverse | 5’-TTCTCGGGCATATTTTGCTGC-3’ |
| ***At4g17470*** | Forward | 5’-CAAAGGTCTGCTCTCCTTGTGA-3’ |
|  | Reverse | 5’-GGAGTGGTTGATGAGGAACTGT-3’ |
| ***At2g45220*** | Forward | 5’-ATGGTGCAGCCAAACTCCAA-3’ |
|  | Reverse | 5’-TAGTGTGAACGCGTGGGTTTT-3’ |
| ***At1g67750*** | Forward | 5’-TTCTCCGACCTTCATTGCCTC-3’ |
|  | Reverse | 5’-TTTCTCCCATTTCGGGTCGC-3’ |
| ***EF-1α*** | Forward | 5’-TCACCCTTGGTGTCAAGCAGAT-3’ |
|  | Reverse | 5’-CAGGGTTGTATCCGACCTTCTT-3’ |

## Supplementary Table S2: Primers used for RT-qPCR experiments.

Supplementary file 1: List of Differentially Expressed Genes (DEGs) between samples

Supplementary file 2: List of GO terms enrichment for DEGs sets

Supplementary file 3: PKL and PKR2 sequence alignment file

Supplementary file 4: PKR2 tree with sequences used for the PAML analysis

Supplementary file 5: Codon alignment of PKR2 sequences

Supplementary file 6: PAML output file for PKR2 (alternate model)

Supplementary file 7: PAML output file for PKR2 (Null model)

Supplementary file 8: PAML output file for PKL
